# Exploring timing activation of functional pathway based on differential co-expression analysis in preimplantation embryogenesis

**DOI:** 10.18632/oncotarget.12339

**Published:** 2016-09-29

**Authors:** Yongchun Zuo, Guanghua Su, Shanshan Wang, Lei Yang, Mingzhi Liao, Zhuying Wei, Chunling Bai, Guangpeng Li

**Affiliations:** ^1^ The Key Laboratory of Mammalian Reproductive Biology and Biotechnology of the Ministry of Education, College of Life Sciences, Inner Mongolia University, Hohhot 010021, China; ^2^ College of Computer Science, Inner Mongolia University, Hohhot 010021, China; ^3^ College of Bioinformatics Science and Technology, Harbin Medical University, Harbin 150081, China; ^4^ College of Life Sciences, Northwest A&F University, Yangling, Shanxi 712100, China

**Keywords:** gene co-expression analysis, embryo early development, dynamics activation, KEGG functional pathway

## Abstract

Recent genome-wide omics studies have confirmed the early embryogenesis strictly dependent on the rigorous spatiotemporal activation and multilevel regulation. However, the full effect of functional pathway was not considered. To obtain complete understanding of the gene activation during early development, we performed systematic comparisons based on differential co-expression analysis for bovine preimplantation embryo development (PED). The results confirmed that the functional pathways actively transcribes as early as the 2-cell and 4-cell waves, which Basal transcription factor, Endocytosis and Spliceosome pathway can represent first signs of embryonic activity. Endocytosis act as one of master activators for uncovering a series of successive waves of maternal pioneer signal regulator with the help of Spliceosome complex. Furthermore, the results showed that pattern recognition receptors began to perform its essential function at 4-cell stage, which might be needed to coordinate the later major activation. And finally, our work presented a probable dynamic landscape of key functional pathways for embryogenesis. A clearer understanding of early embryo development will be helpful for Assisted Reproductive Technology (ART) and Regenerative Medicine (RM).

## INTRODUCTION

The preimplantation studies of mammalian embryos provide key insights into the question of when, where, and how cells take on fate separate [1]. In addition, understanding early development offers a striking opportunity for Assisted Reproductive Technology (ART) [2]. Mammalian preimplantation embryonic development includes a series of important events, such as oocyte maturation, the first cell mitosis, maternal to zygotic transition (MZT), embryonic genome activation (EGA), and cell fate decision [3–5]. All of the above molecular events are critically depended on a strictly controlled system of gene expression [6–8]. Thus, exploring spatiotemporal activation patterns of preimplantation embryos will play important insight into understanding of early developmental mechanism.

In the last decade, advances in sequencing technology, such as multiplex qPCR, microarray, and RNA-seq technology, have accelerated spatiotemporal expression of genes related to embryo development [5, 9, 10]. However, very little progress was obtained owing to the scarcity of early embryos [3, 5, 11]. The first transcriptome profiling of bovine *in vivo* embryos was obtained on Bovine Genome Array [12]. Then, Kues provided the first dynamic transcriptomes of bovine, which included the metaphase II oocytes and every stages of preimplantation embryos [13]. Recently, RNA-seq technology provided two extensive transcriptome dynamics of gene timing activation in bovine PED with the use of *in vitro* embryos [14] and *in vivo* embryos [15]. Both of them provided the largest transcriptome dataset of bovine oocyte maturation and offered detailed information for the timing activation during early embryogenesis. All of the above studies have supported the conclusion that the major maternal to zygotic transition (MZT) begin from 4-cell to 8-cell stages, to which the largest genes of zygotic genome were activated during this period [16, 17]. Gene ontology analysis revealed that these genes are enriched into a series of important biological processes [13, 15, 18].

The patterns of gene expression performed species-specific bias at the important timing wave of genome activation. Xie and co-authors first reported the global transcriptional profiles for mouse, human, and bovine of preimplantation embryo development (PED). Co-expression analysis showed that 40.2% orthologous gene triplets displayed different expression patterns for three mammalian species [19]. Also, based on a more comprehensive stage-specific comparison, Jiang et al. found that mouse, human, and bovine shared more maternal stored genes than EGA genes. They also found that bovine and human embryonic transcriptomes showed more similarity than mouse [14]. Due to the human embryos scarcity, the bovine can be used as one of the best alternative methods for human assisted reproductive technology (ART).

The preimplantation transcriptional organization is highly preserved, and the gene activation is sequentially ordered for preimplantation development [20, 21]. The new challenge is to understand how specific pathway controlling embryonic events and to largely define gene function. Therefore, more insight analyses of full functional pathway throughout preimplantation development are needed to unravel the variation in each of these stereotypic patterns. To fully understand what and when functional pathway can be activated and its further interaction, we delve further into the largest functional pathways of bovine preimplantation embryo development and discuss its timing activation. By comprehensive gene co-expression analysis, the most complete functional pathways related to development from KEGG annotation were first analyzed in this study, including identification of the timing activation patterns of functional pathways and comparisons for different development stage. The results provided a suggested, sequential-order landscape of the molecular pathways for early embryo development.

## RESULTS AND DISCUSSION

### Waves of early transcriptional activation

**Figure 1:**
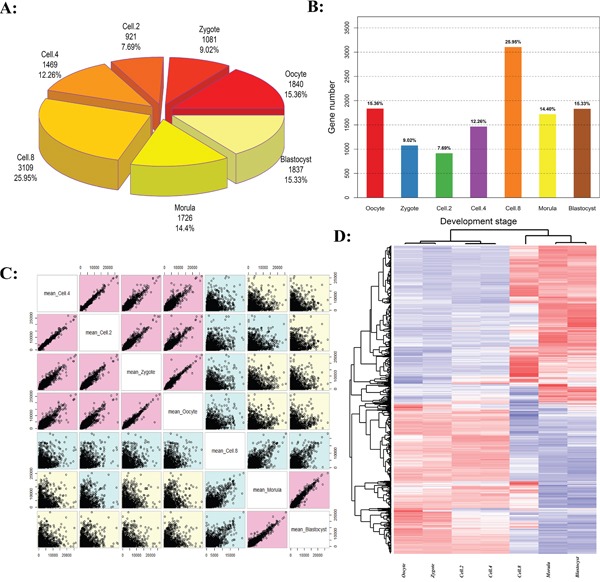
The activation waves of bovine embryo preimplantation development **A** and **B.** The number and proportion of genome activation for different developed stages. **C.** Embryogenesis stages ordered and colored by correlation. **D.** Two-color heatmap representation of maternal-zygotic transition (MZT) transcriptional activation. Hierarchical clustering of the samples was performed using Pearson correlation with average linkage.

The early development of mammalian is strictly dependent on the temporal and spatial specificity wave activation [22]. Figure 1A and 1B give the quantitative proportions of the whole-genome activation at different development stages. The results confirmed that the bovine embryogenesis undergoes successive waves of genome activation. The proportion of expressed activation decreased from 8-cell stage (25.95%) to 2-cell stage (7.69%), as shown in Figure 1A and 1B. The greatest activation wave was found at 8-cell stage (Supplementary Table S1), where 25.95% (3109) genes showed the highest expression, reflecting the important onset of embryonic genome activation (EGA) occurring at the 8-cell stage. The stage pairs comparison demonstrated the transition from maternal genome to embryonic genome (Figure 1C).

The development stages were classified into three groups (Figure 1C): Before Transition (Oocyte, Zygote, Cell-2 and Cell-4), Transitioning (Cell-8, called MZT), and After Transition (Morula and Blastocyst, called EGA) [23, 24]. In the Before Transition stages, the embryo relies on the reserves of proteins and mRNA deposited in the oocyte cytoplasm. In the After Transition stage, the occurrence of EGA marks the beginning of self-sustained cellular biology (Figure 1D, Supplementary Table S2) [25, 26]. During the Transition stage, the gene transcription and translation of the embryonic genome occurs simultaneously, and the majority of oogenetic products are lost due to degradation, including stored maternal RNAs and proteins [27, 28].

### Gene dynamics co-expression of stage bias during *in vivo* preimplantation development

**Figure 2:**
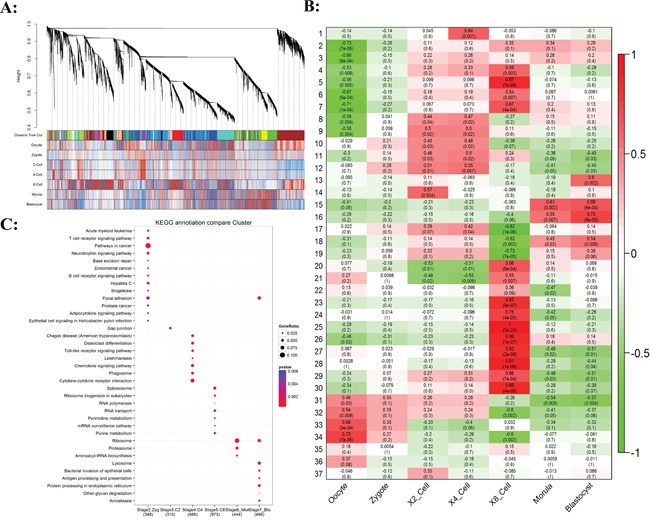
Gene co-expression analysis of stage-specific dynamics during bovine preimplantation development **A.** Hierarchical clustering dendrogram obtained with the weighted correlation network analysis. The first color row underneath (labeled Group) shows the module assignment determined by the Dynamic Tree Cut. The other color rows represent the module location of different development stages. **B.** Stage specific co-expression gene modules and their correlation to development stage. Numbers of each square represent correlation of module and development stage, and p-value of each correlation value. Color of each square is correspond to correlation: Positive correlation (Red); Negative correlation (Green); No correlation (White). **C.** The pathway enrichment of stage-specific significant modules.

Figure 2A showed hierarchical clustering dendrogram for the co-activation pattern of whole genome. The profiles can be clearly clustered into Oocyte/Zygote, 2/4-Cell, 8-Cell, and Morula/Blastocyst patterns. The 8-Cell stage performed the most distinct pattern compared to the other cell types, for which MZT happens at this stage. Based on the weighted gene co-expression network analysis, 37 modules of co-expressed transcripts were identified, in which 4 modules were significant to oocyte/zygote, 7 modules were significant to 2-cell/4-cell, 14 modules were significant to 8-cell, and 4 modules were significant to morula/blastocyst (Figure 2B, Supplementary Figure S1 and Supplementary Table S3-S4). The expression patterns of these modules were mostly well-differentiated among development stages. We then determined the KEGG category enrichment of each module. The Zygote specific module was highly enriched to cancer related signal pathway gene functions (Figure 2C; Supplementary Table S5), including T-cell and B-cell receptor signal pathway. The receptor signal pathway was further increased in the 4-cell stage, which included pattern recognition receptors, Chemokine signal pathway, and Cytokine-cytokine receptor interaction. This indicated that full establish of pioneers signal pathway was a prerequisite for the following MZT wave. The 8-cell specific module contained the main ZGA transcripts, such as Spliceosome, RNA transport, and DNA polymerases.

The degradation of maternal transcripts was enriched in the morula/blastocyst stage. These two stage embryos showed somewhat similar expression profiles. The largest module was enriched for genes involved in Ribosome for both morula and blastocyst stage. It encompasses two major molecular activation groups, the maternal transcripts degradation and the new zygotic procedure installation, with both groups combined can perform reprogramming from differentiated terminal to totipotency. From the result, we can see that maternal transcripts enriched into signal-receptor related pathways and the zygotic transcripts prefer to the essential genes of embryo development. The total numbers of differential expressed gene in each molecule event group did not differ much, but the difference between groups was significant.

### The comparison of oocytes transcriptome for *in vivo* and *in vitro* maturation

**Figure 3:**
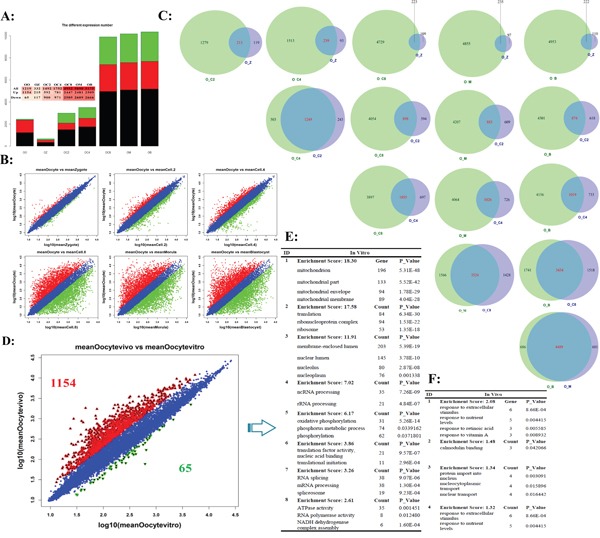
The different expression profiles when using the oocyte's transcriptome as background **A.** The numbers of differential transcript expression for different stages in preimplantation development. **B.** Scatter plot compares the results of log transformed gene expression levels and the differentially expressed gene distribution pattern before MZT (up-line) and after MZT (down-line). The genes were classified into three classes. Red genes are up-regulated that gene expression of right sample is larger than left sample. Green genes are down-regulated that gene expression of left sample is larger than right sample. Blue genes are not differentially expressed genes. **C.** Venn diagram of shared and unique genes between any two develop stages based on oocyte's transcriptome as control. **D.** The transcriptome comparison between *in vivo* and *in vitro* matured bovine oocytes. **E** and **F.** Top functional enrichment for *in vivo* (E) and *in vitro* (F) matured oocytes based on gene ontology, respectively.

The oocyte is the crucial driver for PED [29, 30]. The success of fertilization and the outcome of consequent embryo development is determined by the quality of the matured oocytes [31]. Therefore, it is necessary to further uncover the most representative and correlated gene markers for oocytes matured quality. When using the oocyte as control genome-wide profile pattern of whole gene expression, the total stages of early embryogenesis were clustered into two temporal classes (Figure 3A and 3B, Supplementary Table S6): The first represents the mature oocyte until the late 4-cell embryo, and the second represents the subsequent stages up to the blastocyst stage. The results again revealed the greatest difference occurred at the 4-cell to 8-cell stages (MZT), the largest transition of differentially regulated genes were identified from hundreds to thousands (Figure 3B). The oocyte profile pattern of whole gene expression represented the maternal transcriptome. Based on oocyte's transcriptome as control, the Venn diagram showed shared and unique genes between any two develop stages (Figure 3C). We observed that there was extensive overlap in either Before Transition stages or After Transition stages, but also shown significant differences. For example, oocyte_vs_4-cell (O_C4) and oocyte_vs_2-cell (O_C2) shared the most similar patterns of differential gene expression, and the oocyte_vs_blastocyst (O_B) and oocyte_vs_morula (O_M) shared the largest overlap during After Transition stages (Figure 3C).

It is commonly believed that oocytes matured *in vivo* superior to *in vitro* maturation oocytes [32]. Even with continuous improvement, the comparable oocytes still had been not obtained. The comparison of the transcriptomic profile matured *in vivo* and *in vitro* demonstrated this conclusion. In oocytes matured *in vivo*, cutoff of fold change is 2, more than 1,000 genes were filtered compared to those matured *in vitro* (Supplementary Table S7). The GO enrichment analysis showed that these up-regulated genes were enriched in mitochondrion, ribonucleo protein, oxidative phosphorylation, and so on (Figure 3E) [33]. *In vitro* matured oocytes were more susceptible than *in vivo* matured oocytes, this can be confirmed in the up-regulated genes involved in the calmodulin binding and “response to” related genes (Figure 3F, Supplementary Table S8). Data from the current analysis suggested that although *in vitro* maturation oocytes nearly resembled *in vivo* maturation oocytes in term of nuclear maturity, several pathways associated with energy accumulation preferred to up-expressed in an immature manner (Supplementary Figure S2). These differentially expressed genes/pathways provide evidence for *in vitro* maturation (IVM) optimization.

### Spatiotemporal expressed profiles of genome-wide transcripts for preimplantation embryos development

**Figure 4:**
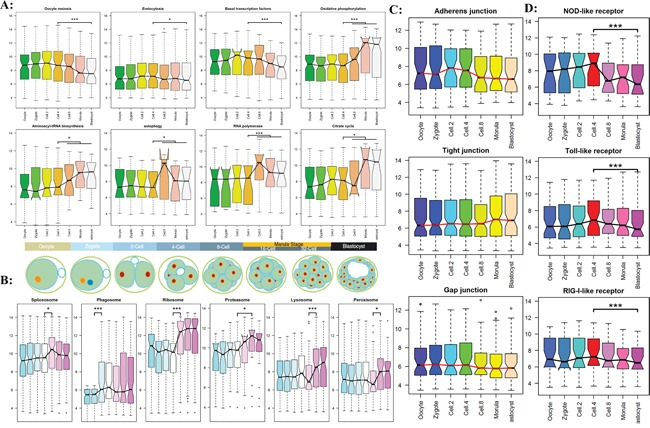
The timing genome-wide activation of KEGG functional pathway in preimplantation embryogenesis. The results of RNA-seq experiment were shown in supplementary Figure S5-S8 **A.** The dynamic patterns of the important biology pathways for different development stages. **B.** The sequential order expression of organelle related functional pathway. **C.** The dynamic expression of three cell junction families. **D.** The dynamic expression of three receptor families.

To better understand what and when functional pathways join in regulation, the most comprehensive genome-wide activation map of functional pathways should be established. Based on the modules of gene co-expression analysis, 34 functional pathways and 26 Signal transductions were downloaded from the KEGG website (Supplementary Figure S3, S4). By integrating 4 Receptor pathways and 6 Histone related pathways, a total of 70 functional pathways for all preimplantation development stages were first analyzed in this study. The results in Figure 4 confirmed the bovine embryos undergo a successive time-ordered activation of key functional pathways, including the most important biological pathways (Figure 4A, Supplementary Figure S5), organelle related functional pathway (Figure 4B, Supplementary Figure S6), junction pathways (Figure 4C, Supplementary Figure S7), and receptor pathways (Figure 4D, Supplementary Figure S8). They depended on a tightly controlled and well-orchestrated program. To further prove this conclusion, we analyzed the latest to date public RNA-seq data of bovine *in vivo* preimplantation development from GEO dataset. The dataset consisted of 8 stages of embryos development, the result again demonstrated conclusions (Supplementary Figure S5-S9).

**Figure 5:**
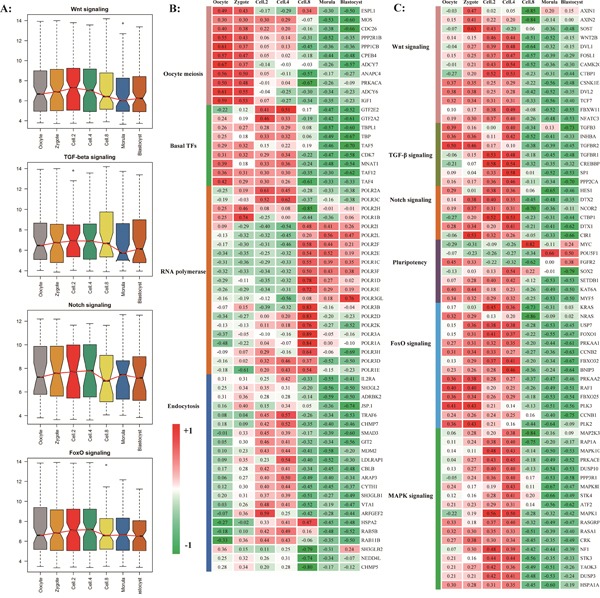
The timing expression of maternal pioneer master regulator **A.** The pre-activation of key signal pathways before major waves of embryonic genome activation. The results of RNA-seq experiment were shown in supplementary figure S9. **B** and **C.** The significantly genes from housekeeping pathways (B) and pioneer signal pathways (C).

The timing analyses of genome-wide transcripts uncovered a series of sequential order waves during early embryogenesis. We found the embryonic transcriptional activation begins as early at the 2-cell stage [34, 35]. The minor wave involved in essential signaling and metabolic pathway were consequently activate, including MAPK, Wnt, TGF-beta, Notch etc. (Figure 5A, Supplementary Figure S9). This so-called early rise requires specific transcriptional regulators, such as transcription factor and RNA splicing complex subunit. In addition, the dynamic activation of transcripts related to pluripotency regulation was analyzed (Figure 5B and 5C, Supplementary Figure S10). We found that SOX2 take part in the initial embryo cleavage along with SMAD, STAT, BMP transcription factors, OCT4, KIF4 and c-Myc were activated at blastocyst stages.

The Endocytosis and Basal transcription factors as potential master regulator became transcriptional activation and began to contribute to the earliest development process while the transcripts involved in oocyte meiosis are degraded after fertilization (Figure 5B). Three families of pattern recognition receptors performed the essential function at 4-cell stage (Figure 4B). At MZT stage, for the upregulated genes, we found a clear enrichment of gene ontology classification whose transcripts are involved in RNA processing, RNA splicing, ribonucleoprotein complex biogenesis, and ribosome biogenesis, indicating that the major waves of embryonic specific transcripts are initiated and translation machinery is establishing [17, 36].

### The probable timing activation landscape of functional pathway modulating by Endocytosis in mammalian early embryogenesis

**Figure 6:**
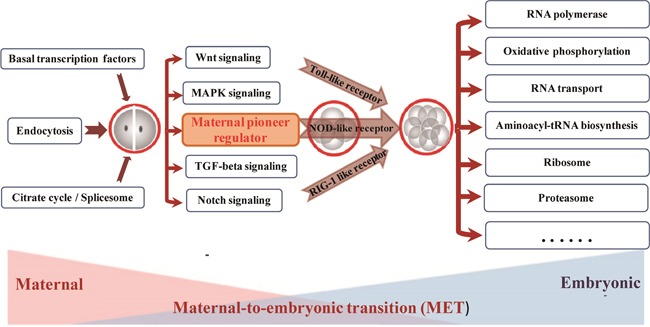
The probable timing activation of functional pathway during bovine preimplantation development

In this study, we aimed to reveal the early embryogenesis depends on what genetically encoded events. Recent findings have provided cleared evidence for our understanding of molecule mechanisms in PED, but not as clear at functional pathway level. The comprehensive transcriptional analyses in this study provided a probable landscape of functional pathway activation for bovine early embryonic development (Figure 6).

The early embryo development is governed by a highly hierarchical program of molecular and cellular events, mainly encompasses two major molecular activities: maternal clearance and zygotic genome installation (Figure 6). The Basal transcription factors, Endocytosis, Spliceosome pathway etc are belonging to this phase of preimplantation development. These mRNAs usually deposited in message ribonucleoprotein (mRNP) complexes, and localized in the cytoplasm or dispersed within the cytoplasm. By receptor-mediated endocytosis, the Endocytosis pathway plays a transfer role in specifically activate, promote, and degrade the signal pathway during mammal embryonic development [37, 38]. With the help of spliceosome complex and several key signal pathways as pioneers, Wnt, Notch, TGF-beta etc were activated to uncover a series of successive waves of embryonic genome activation that occur as early as the 2-cell stage [5, 39, 40].

Establishing critical levels of pioneers signal pathway plays a significant role for major ZGA wave, which may further extensively activate three pattern recognition receptors at 4-cell stage [6]. For example, previous studies reported the Toll receptors are essential for Drosophila embryonic development and immunity [41]. As development proceeds, the development control will be shifted from maternal program to zygotic transcripts at 8-cell stage, which is referred to as the maternal to zygotic transition (MZT). The largest number of embryonic genome transcripts activation (EGA) is initiated to begin self-sustained cellular biology. The functional pathways involve in RNA polymerase, Oxidative phosphorylation, RNA transport, ABC transporters, Ribosome, and Proteasome [20, 21]. Together, the series of activation waves dramatically remodeled the landscape and cellular identity of embryonic gene activation in mammalian early embryo development.

## MATERIALS AND METHODS

### Data collection and comparison

The gene expression datasets of bovine preimplantation embryos were downloaded from NCBI's Gene Expression Omnibus [42]. They contained the 23 microarray samples of bovine and 16 RNA-seq samples of bovine *in vivo* preimplantation embryos. They were deposited under accession number GSE12327 and GSE36552, respectively. They are include oocyte stage, zygote stage, 2-cell stage, 4-cell stage, 8-cell stage, morulae stage, and blastocyst stage, Each stage was composed of 2-4 biologically replicated samples. High pearson correlation coefficients were found among biological replicates of the same developmental stage (Supplementary Table S9, Supplementary Figure S11). Each gene symbol of the whole profile was mapped to its corresponding the gene symbols, and that have no corresponding gene annotation were discarded to reduce the potential noise. Lastly the expression profiles in each sample were processed by quantile normalization that accounts for different amounts of RNA present throughout embryo early development.

For the datasets comparison, the same functional pathways were downloaded from the KEGG website. Then the official gene symbols were extracted from each transcriptome list. Because the difference of sequencing platform, the detected transcripts from RNA-seq transcriptome are more than microarray. Our comparison results confirmed the statistical significance of every pathway were stable for different development stages. The boxplot was used to character comprehensive difference of gene expression, including median, range, outliers and distribution of one dataset. The line graph of median was integrated to reflect the activation trend of functional pathways in preimplantation development. The biological significance of every pathway was calculated by using the T test validation.

### Weighted gene co-expression network analysis (WGCNA)

In this study, the weighted gene co-expression network (WGCNA) approach was initially employed to construct the network [43]. This approach has been widely employed to construct gene modules within a network based on correlations in gene expression. A blockwise Modules R function was implemented using the following parameters: power 5 9, minModuleSize 5 20, deepSplit 5 0, neworkType 5 “signed”. Briefly, Pearson correlation coefficients were calculated for all pair-wise comparisons of the genes across all samples. The resulting Pearson correlation matrix was transformed into an adjacency matrix by a power function, which resulted in a weighted network. Topological overlap measure (TOM) was then calculated using a dynamic tree-cutting algorithm. Genes were hierarchically clustered using 1-TOM as the distance measure and modules were determined by choosing a height cutoff 0.995 for the resulting dendrogram. The module eigengene (ME) corresponds to the first principal component of a given module. It can be considered as the most representative gene expression in a module. Module membership (MM) for each gene in each module refers to the Pearson correlation between the expression level of the gene and the ME.

### Identification of gene co-expression network construction by WGCNA

To obtain further understanding of whether these gene co-expression waves correlated with time-specific development stage, we performed gene co-expression analysis for individual embryo types [43]. Based on pickSoftThreshold function, the soft-thresholding power was performed to choose a proper soft-thresholding power (Supplementary Figure S12). Then average linkage hierarchical clustering was performed on the dissimilarity matrix and the clustering tree (dendrogram) was formed (Figure 2A). In the dendrogram, the nodes in bottom row represent the genes, and the nodes in other rows represent the clusters to which the genes belong, with the branches connecting the nodes representing the distances (dissimilarities). The distance between merged clusters is monotonically decreasing with the level of the splitting: the height of each node is the intergroup dissimilarity (two genes with exactly the same expression pattern across samples will have heights of zero).

### Functional annotation of modules

Annotation of network modules was performed using the Database for Annotation, Visualization and Integrated Discovery (DAVID) with the background list of all genes on the array [44]. In DAVID, an over representation of a term is defined as a modified Fisher's exact P value with an adjustment for multiple tests using Benjamini method. Data analysis of biological significance and visualization were done using R.

## CONCLUSIONS

Mammalian PED is a complex process including sequential ordered activation. All of previous gene expression profiling were limited to simple enrichment analysis of differentially expressed genes, while not considering the full functional pathway effect. A comprehensive analysis for 80 functional pathways brings striking opportunity to fully understand what and when functional pathways activation, and to identify which potential master regulators for cell fate decision. The results confirmed the bovine embryo undergoes a successive time-ordered activation of key functional pathways, which depends on a strictly controlled and thoroughly coordinated system. These gene transcripts, vigorously transcribed at the 2-cell and 4-cell stages, should act as earlier signs of EGA, which might be required to coordinate later major activation. We found that each developmental stage can be described succinctly by small group of functional modules of co-expressed genes, for which they indicate a sequential order of transcriptional changes in pathways of cell cycle, gene regulation, translation, and metabolism, thus outlining the stages from cleavage to morula. The results provided important evidence to uncover molecular mechanisms underlying progressive development of mammalian PED and should offer useful insights in the identification of healthy embryos. Improvement of the assisted reproductive technologies (ART) promotes reproduction in human, livestock, and endangered species to ensure their survival.

## SUPPLEMENTARY MATERIALS FIGURES AND TABLES




















