# Supplementary material for: Exploring timing activation of functional pathway based on differential co-expression analysis in preimplantation embryogenesis
Source: Oncotarget. 2016 Sep 29;7(45):74120–31. doi: 10.18632/oncotarget.12339 (PMC5342040; doi:10.18632/oncotarget.12339)
Supplement: Supplementary file 1 [file oncotarget-07-74120-s001.pdf]

Exploring timing activation of functional pathway based on differential co-expression analysis in preimplantation embryogenesis

SUPPLEMENTARY FIGURES AND TABLES

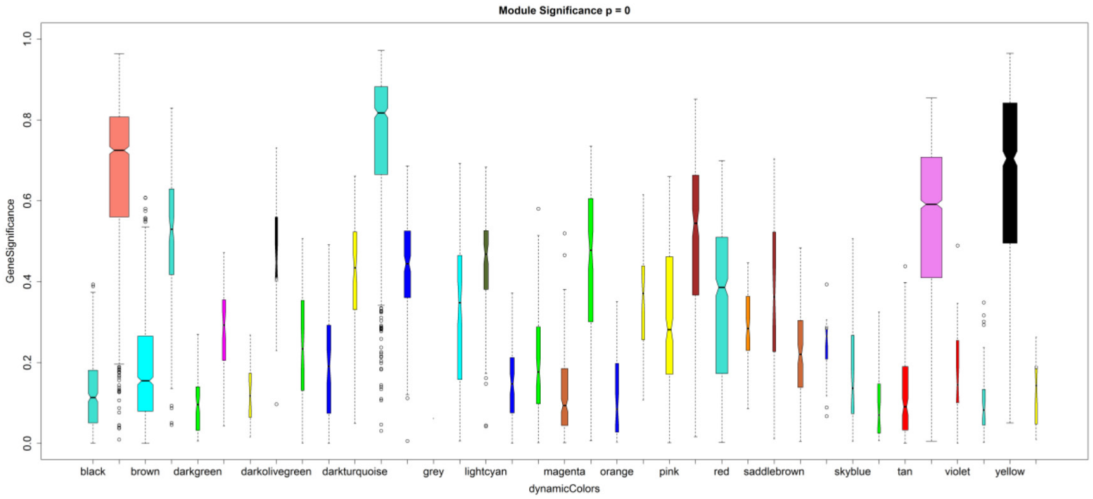

Supplementary Figure S1: The verbose boxplot of module significance.

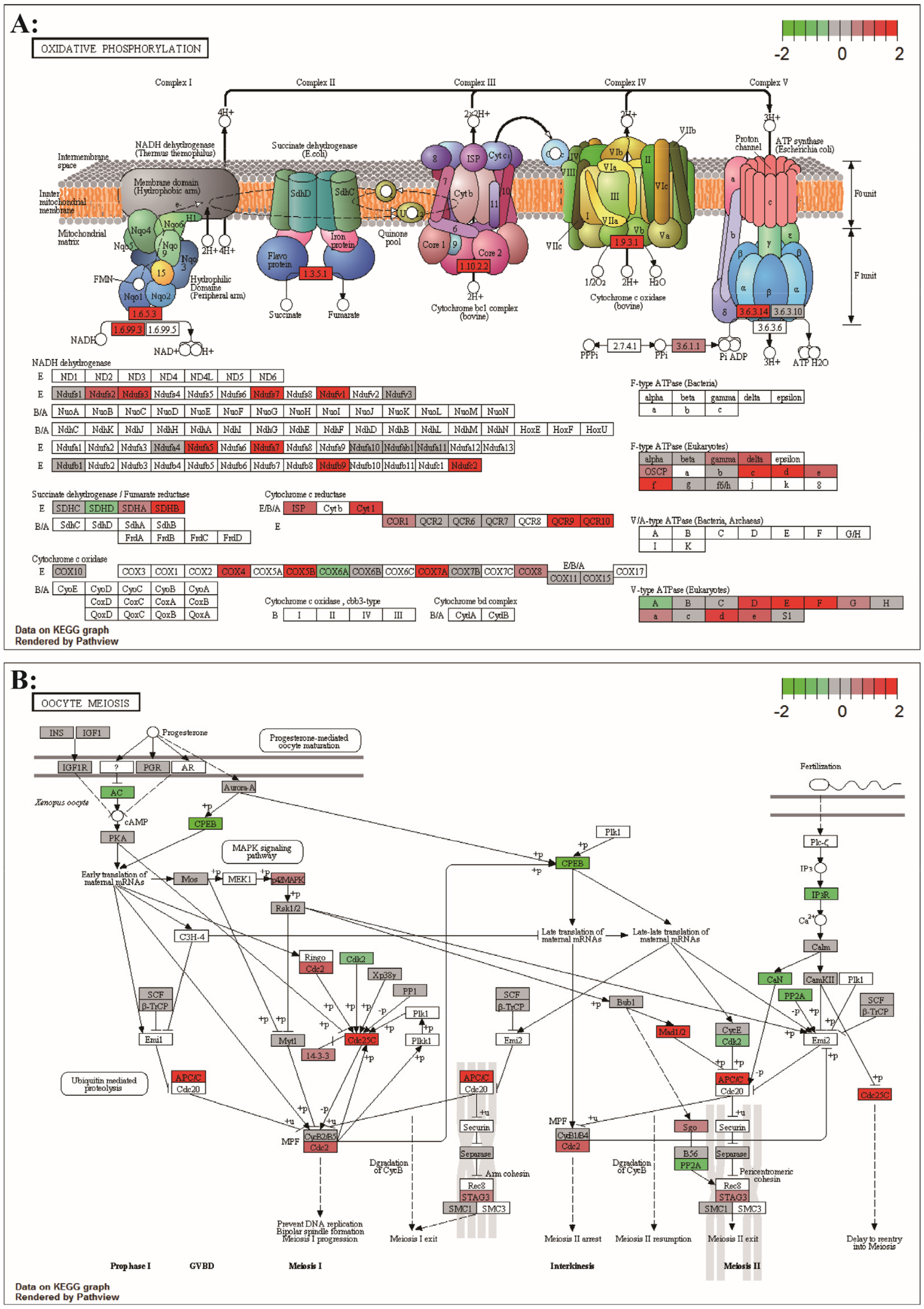

Supplementary Figure S2: The expression of oxidative phosphorylation pathway (A) and oocyte meiosist (B) when *in vivo* compared to *in vivtro* maturated oocyte.

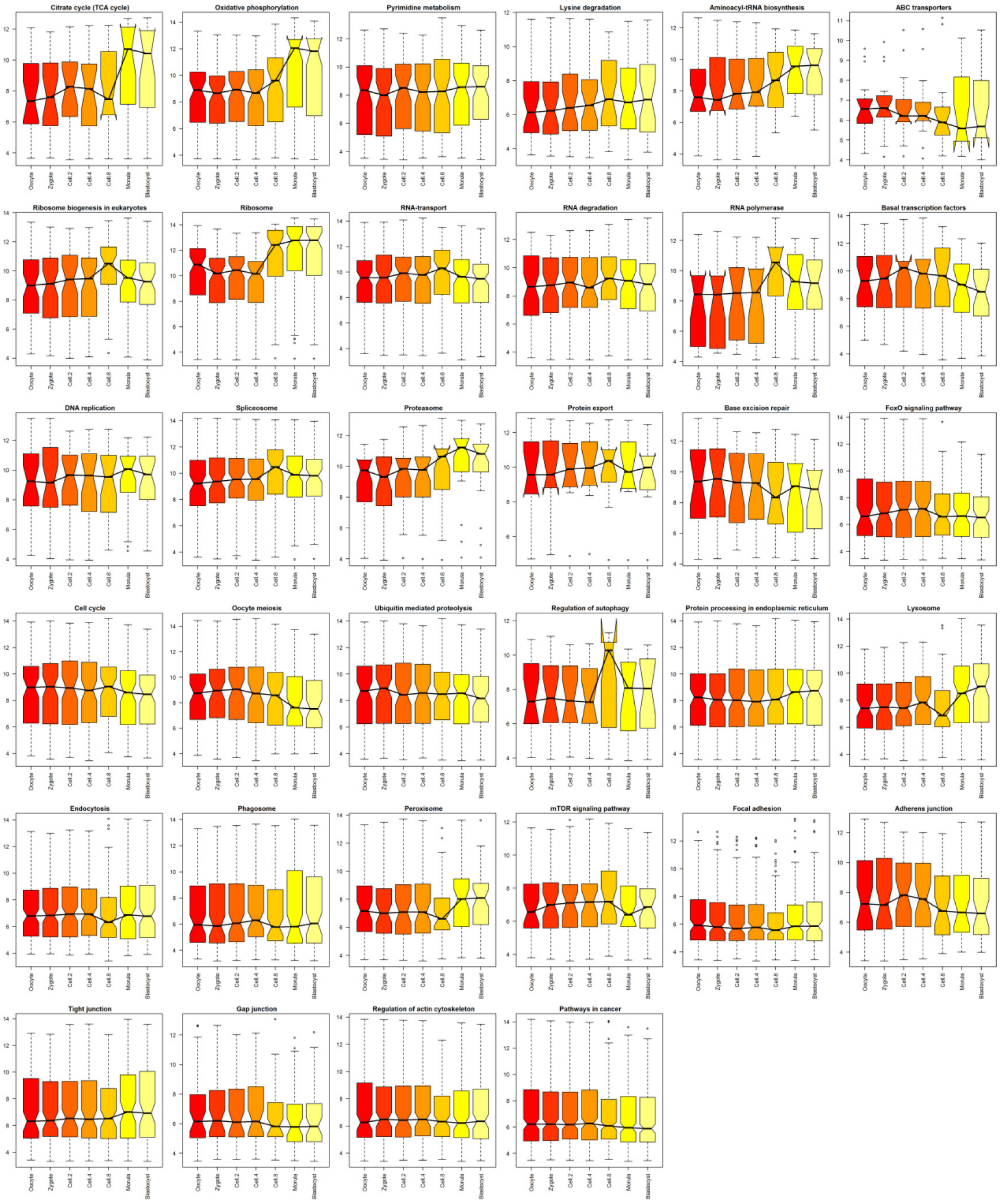

Supplementary Figure S3: The dynamic patterns of all the 34 biology pathways for different development stages.

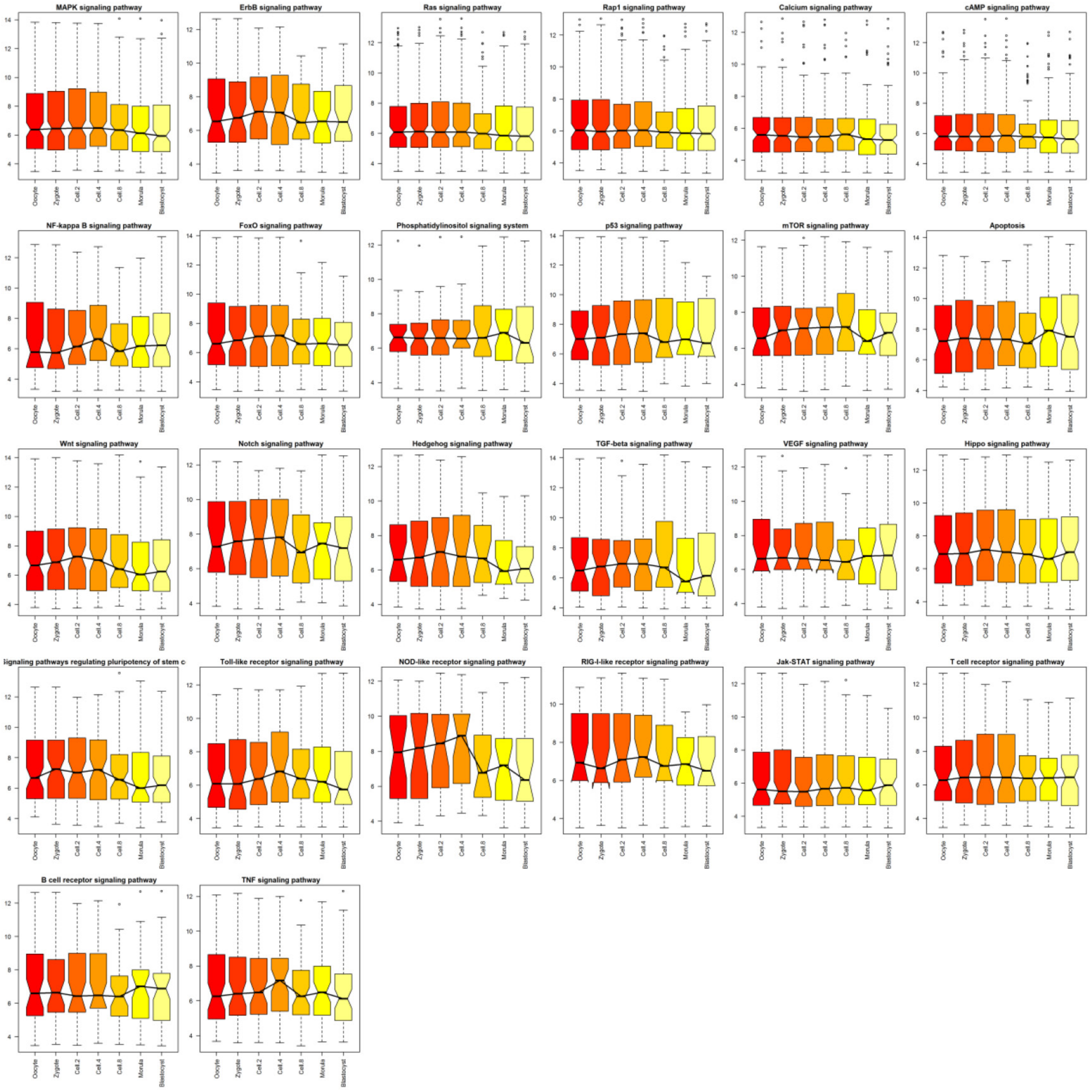

Supplementary Figure S4: The dynamic patterns of all the 26 signaling pathway for different development stages.

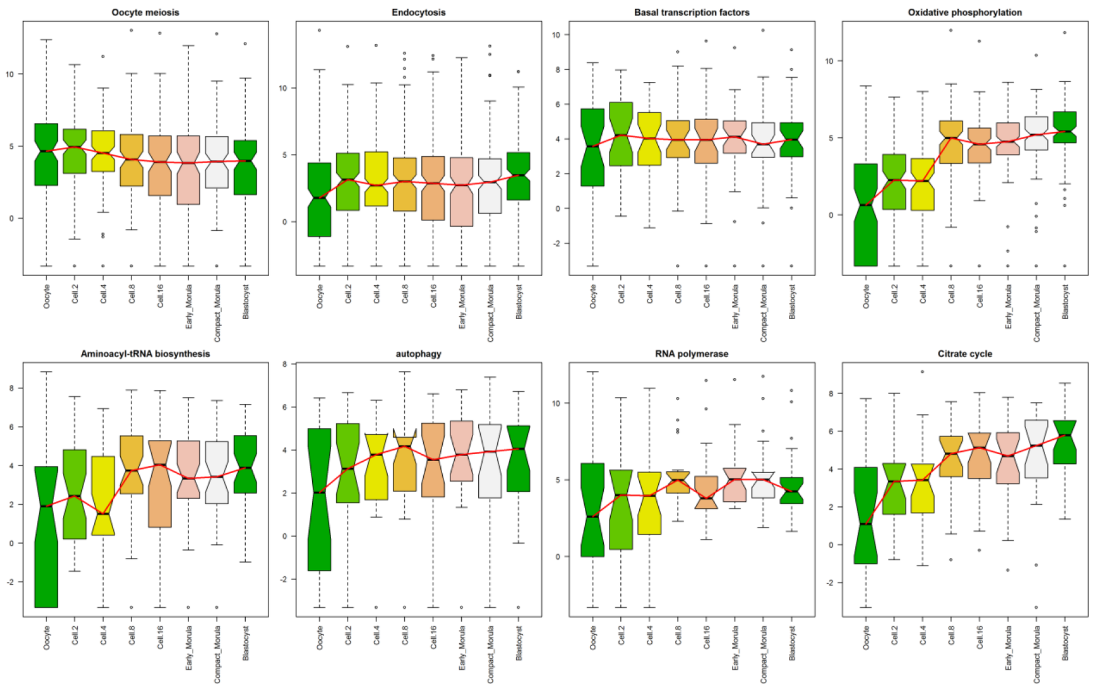

Supplementary Figure S5: The dynamic patterns of the important biology pathways for different development stages based on RNA-seq experiment.

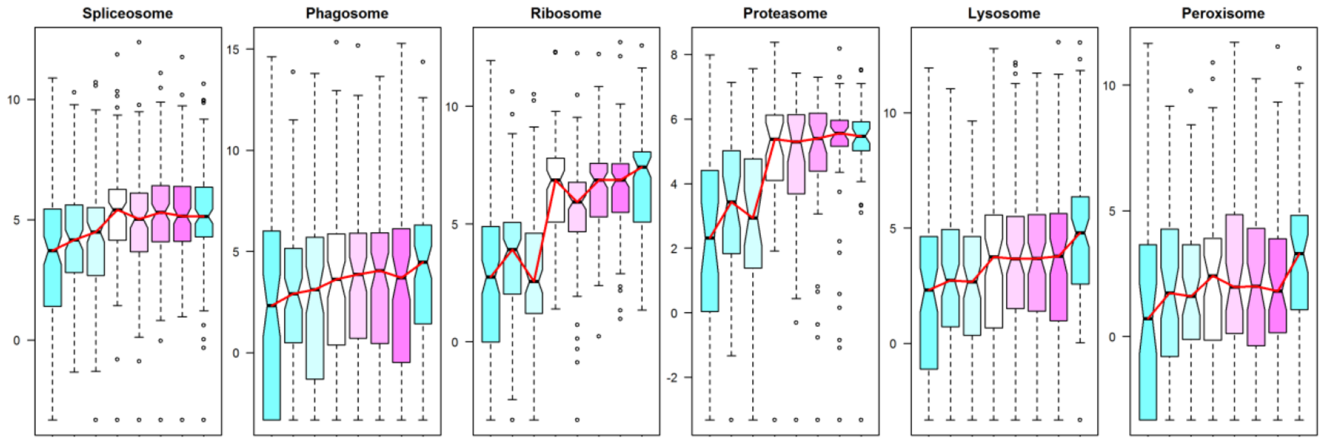

Supplementary Figure S6: The sequential order expression of organelle related functional pathway based on RNA-seq experiment.

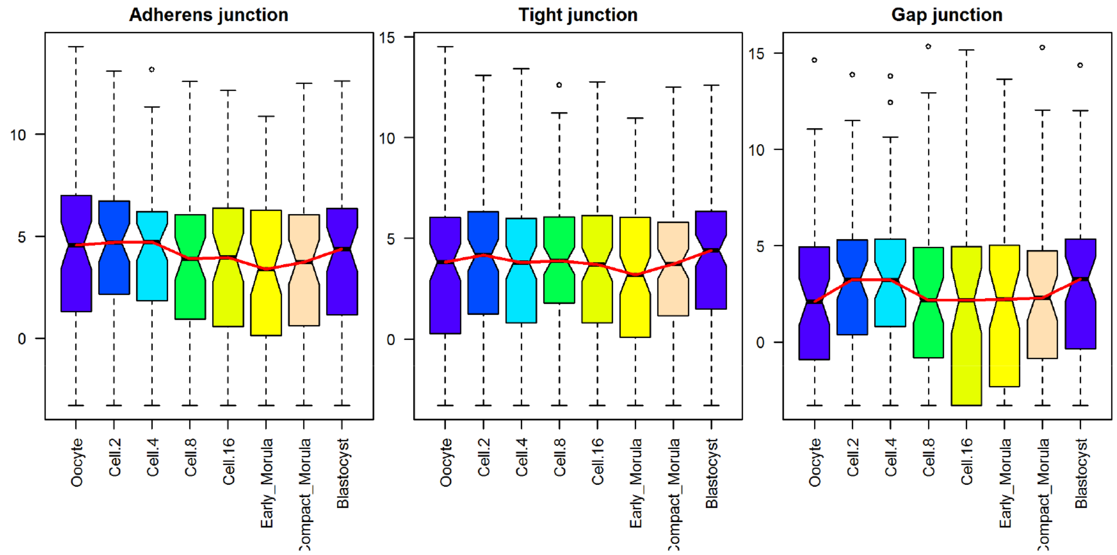

Supplementary Figure S7: The dynamic expression of three cell junction families based on RNA-seq experiment.

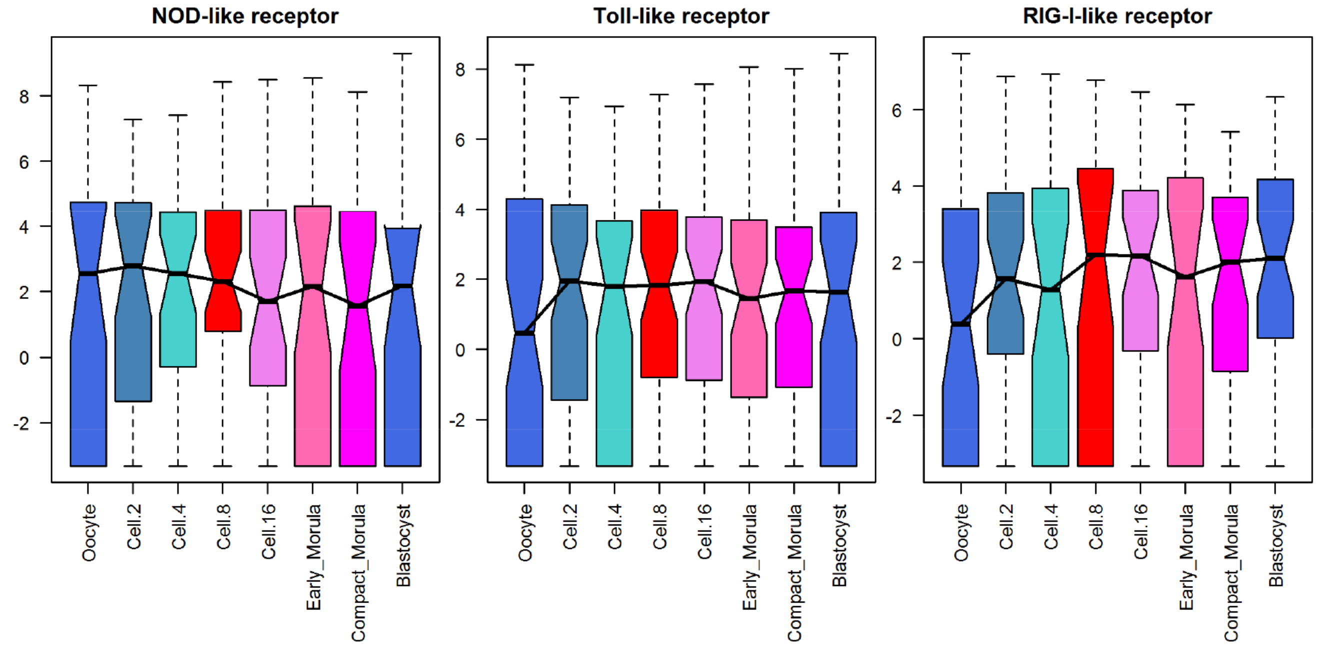

Supplementary Figure S8: The dynamic expression of three receptor families based on RNA-seq experiment.

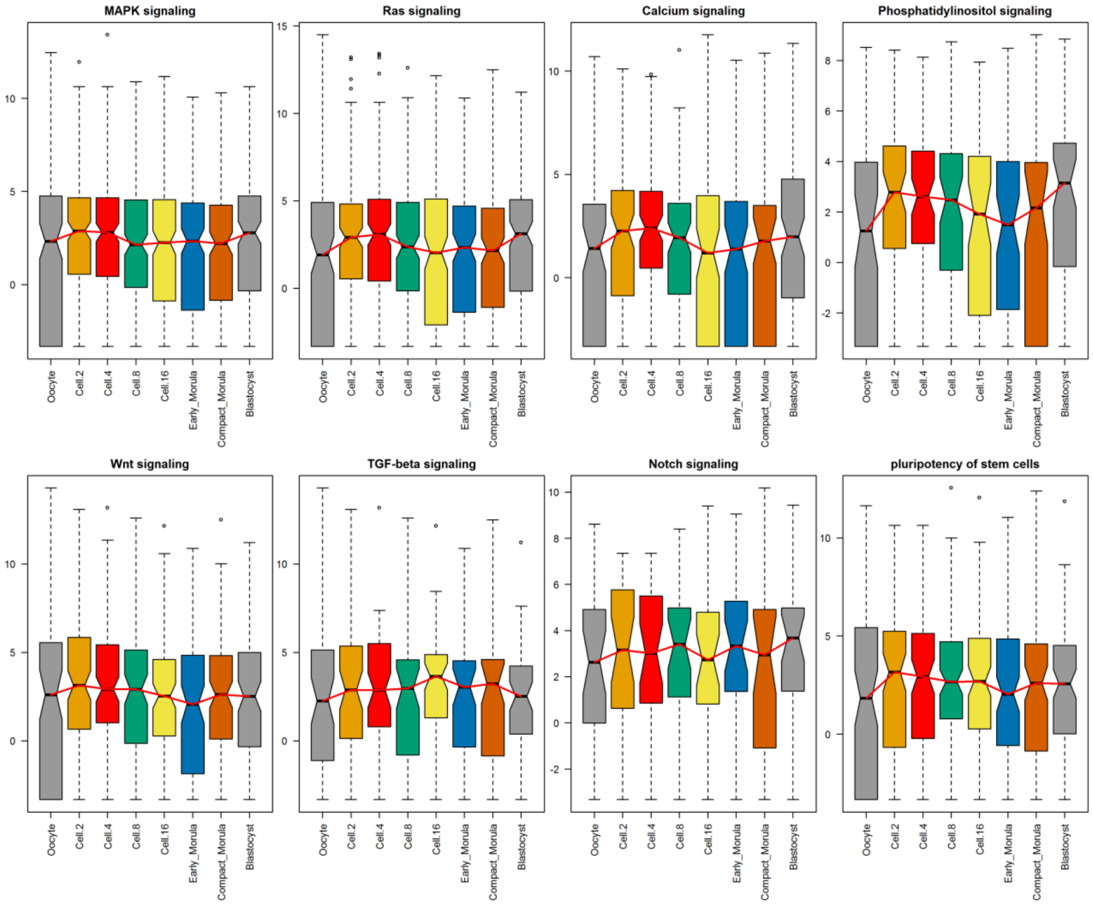

Supplementary Figure S9: The pre-activation of key signal pathways before major waves of embryonic genome activation. based on RNA-seq experiment.

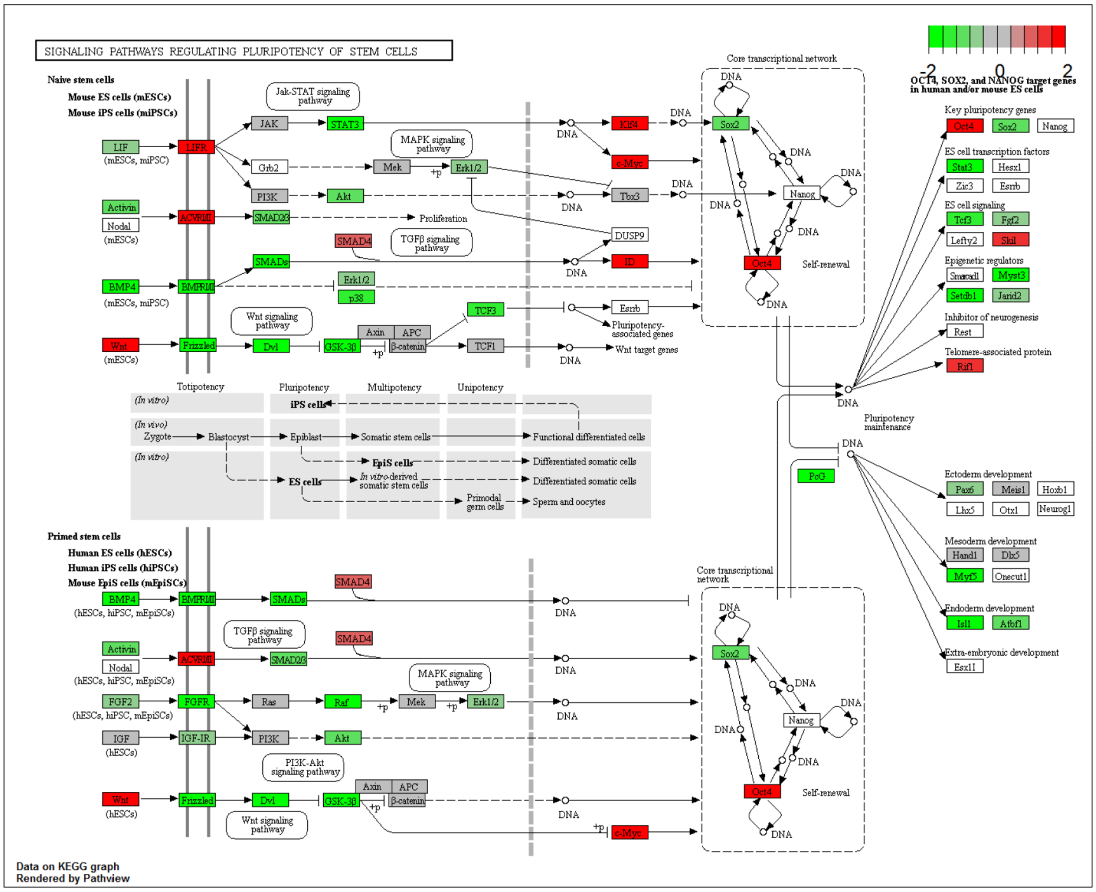

Supplementary Figure S10: The expression regulation of pluripotency of stem cells when Blastocyst embryo compared to Oocyte. The KEGG accession number:bta04550.

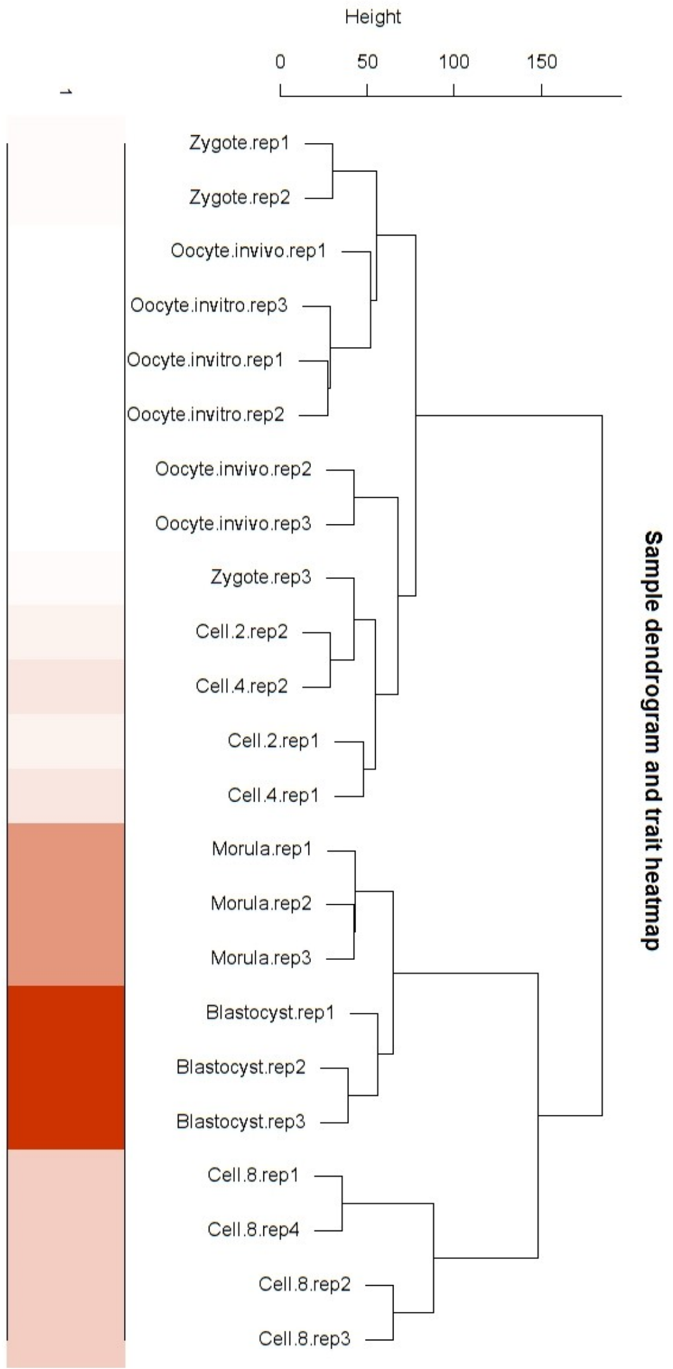

Supplementary Figure S11: Unsupervised clustering of the transcriptome of known RefSeq genes during the eight consecutive stages of bovine preimplantation development.

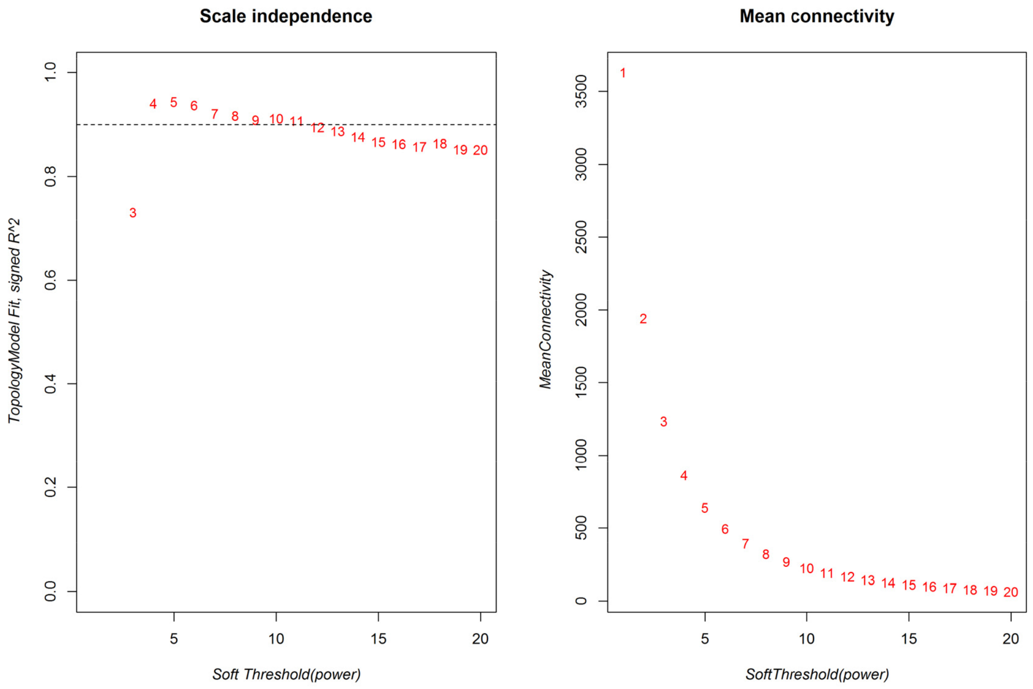

Supplementary Figure S12: The soft-threshold power for analysis of network topology.

Supplementary Table S1: Activation waves of early embryonic development

See Supplementary File 1

Supplementary Table S2: The significant differentially expressed genes between Oocyte and Morula cells

See Supplementary File 2

Supplementary Table S3: P Value and correlation of modules by using WGCNA method

See Supplementary File 3

Supplementary Table S4: The Modules of co-expression genes calculated by using WGCNA

See Supplementary File 4

Supplementary Table S5: The function enrichment of significant modules for different development stages

See Supplementary File 5

Supplementary Table S6: The result of differentially expressed genes between oocyte and other development stages

See Supplementary File 6

Supplementary Table S7: The differentially expressed genes between *in vivo* and *in vitro* maturated oocyte

See Supplementary File 7

Supplementary Table S8: The DAVID GO enrichment of differentially expressed genes between *in vivo* and *in vitro* maturated oocyte

See Supplementary File 8

Supplementary Table S9: The samples used in this study

See Supplementary File 9
